# Supplementary material for: Distinct mutational signatures characterize concurrent loss of polymerase proofreading and mismatch repair
Source: Nat Commun. 2018 May 1;9:1746. doi: 10.1038/s41467-018-04002-4 (PMC5931517; doi:10.1038/s41467-018-04002-4)
Supplement: Supplementary file 1 — Supplementary Information [file 41467_2018_4002_MOESM1_ESM.pdf]

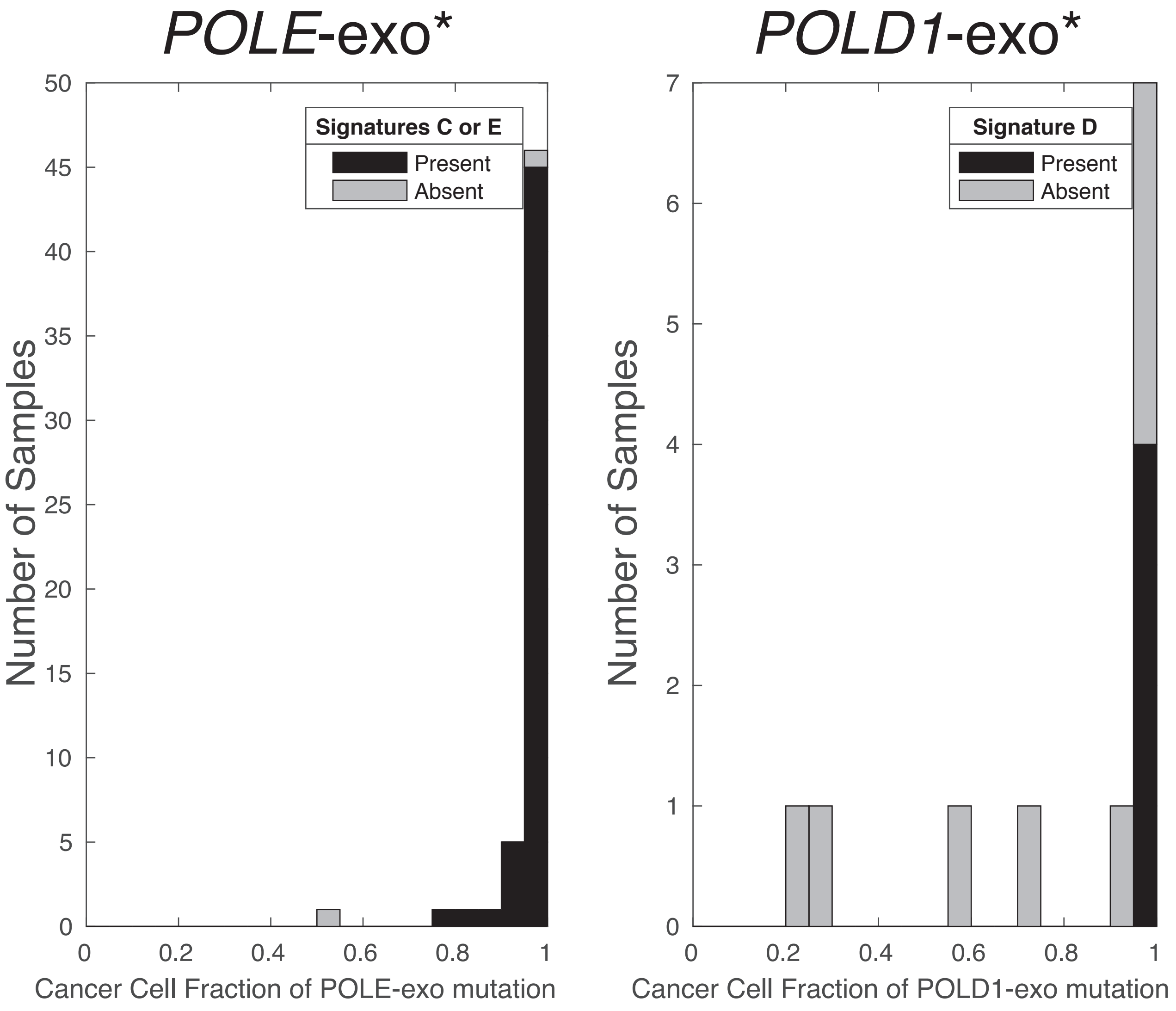

Supplementary Figure 1. Clonal POLE/POLD1 mutations are associated with POLE/POLD1 mutational signatures. For all samples with a POLE or POLD1 exonuclease domain mutation, the fraction of tumor cells in the sample (the cancer cell fraction, CCF) that possess the POLE/POLD1 exonuclease mutation was inferred (Methods). Clonal mutations that are present in most cancer cells (i.e., high CCF) are likely to be driver mutations whereas subclonal mutations (low CCF) are more likely to be passenger mutations. Bars are shaded black for samples with major mutational contributions (>35% of total mutation burden) from Signatures C/E and D in POLE and POLD1 mutated tumors, respectively. POLE/POLD1 exonuclease domain mutations with lower cancer cell fractions (i.e., non-clonal) typically lack of a dominant POLE or POLD1 signature, suggesting that these POLE/POLD1 mutations may not impact polymerase proofreading.

Mutational Signatures in 531 Endometrial Cohort

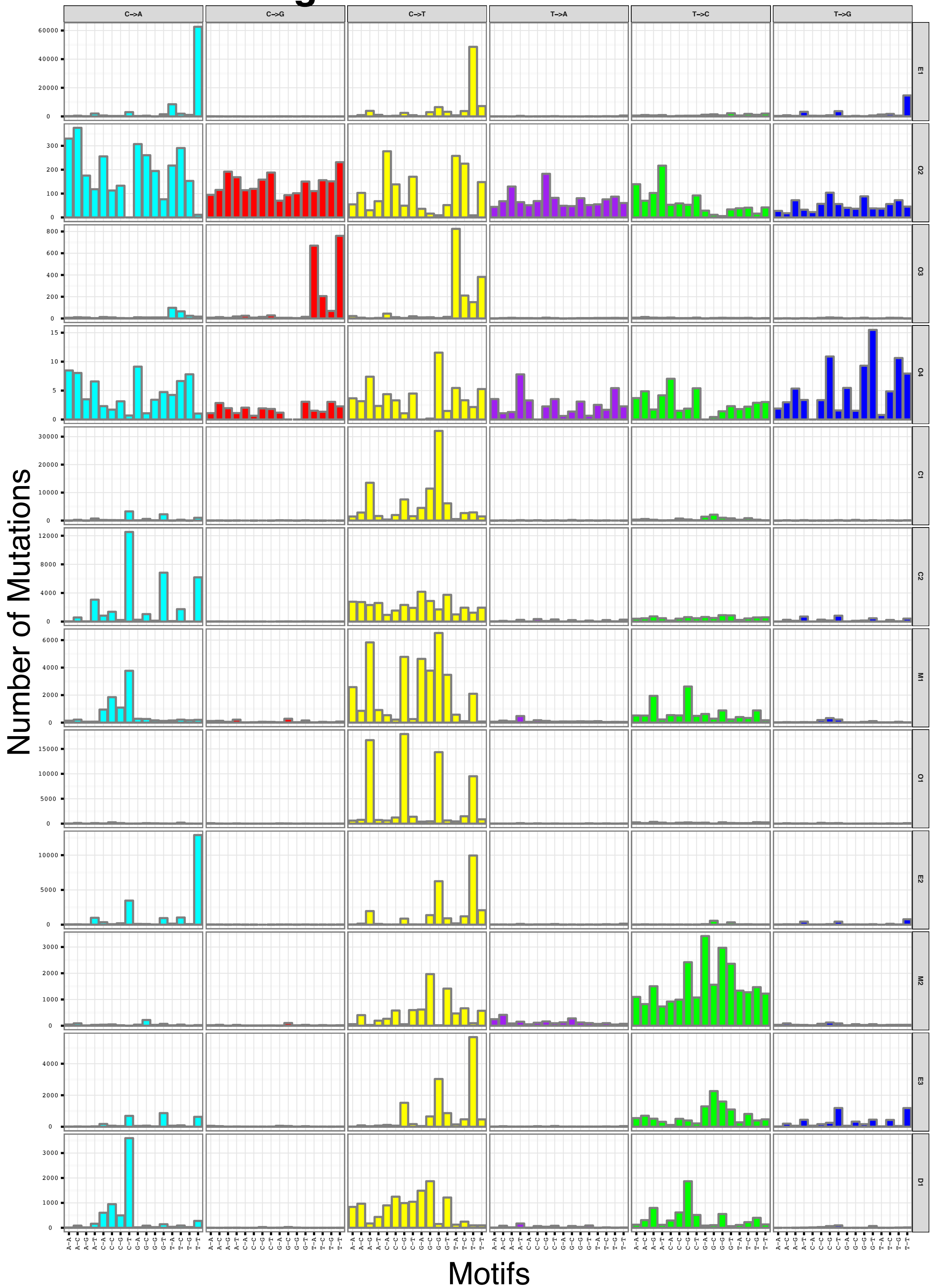

Supplementary Figure 2. Twelve unique mutational signatures were identified in the TCGA endometrial cancer cohort (n=531). Signatures O1-4 are not specifically enriched in samples with polymerase proofreading or MMR deficiency. Signature O1 has been associated with CpG deamination, corresponding to COSMIC signature 1. This signature is also present with large numbers of mutations in MSI samples. Signature O2 is associated with deficiency in the homologous recombination pathway, analogous to COSMIC Signature 3. Signature O3 is associated with APOBEC family deaminase activity, a combination of COSMIC Signatures 2 and 13. Signature O4 comprises primarily insertions and deletions with lower levels of SNVs. It does not correspond to a previously reported signature. The C, D, E, and M signatures are discussed at length in the main text and figures.

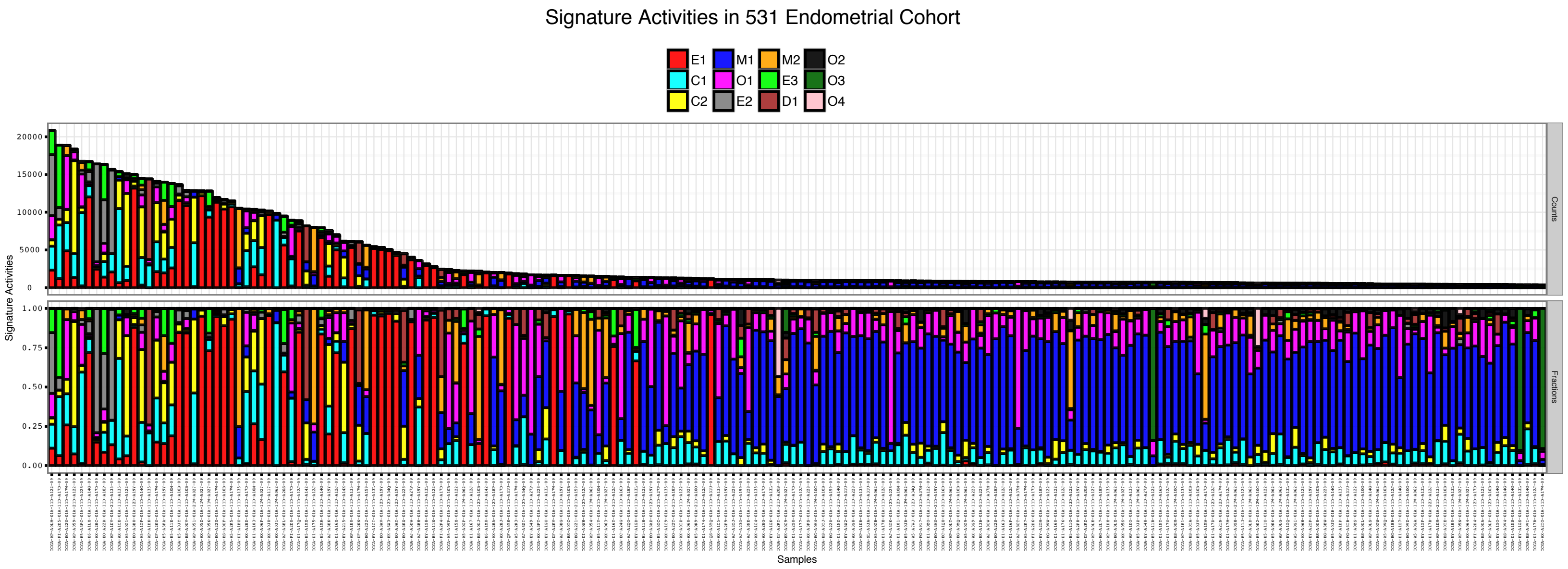

Supplementary Figure 3. Contributions of all 12 mutational signatures across the 200 (of 531) endometrial tumors with the highest mutation burdens. The top panel shows the total number of mutations contributed by each signature and the bottom panel shows the fractional distribution.

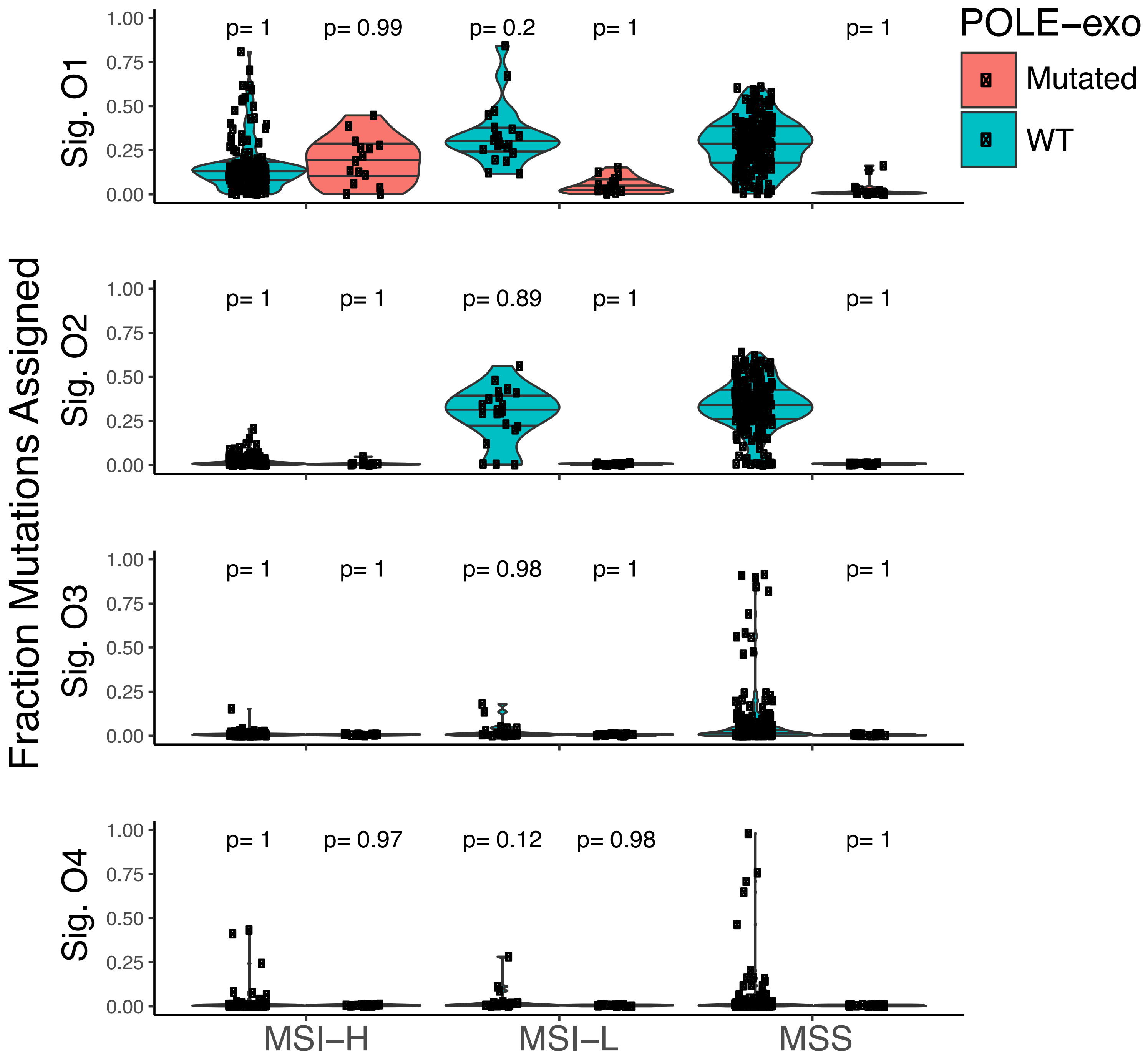

Supplementary Figure 4. Signatures O1-O4 are not associated with *POLE* exonuclease mutations or MSI. The fraction of mutations assigned to Signatures O1-4 are shown for all tumors (segregated by MSI and *POLE* status). P-values represent a one-tailed rank-sum test comparison to MSS tumors lacking *POLE/POLD1* exonuclease mutations, as in Figure 2b.

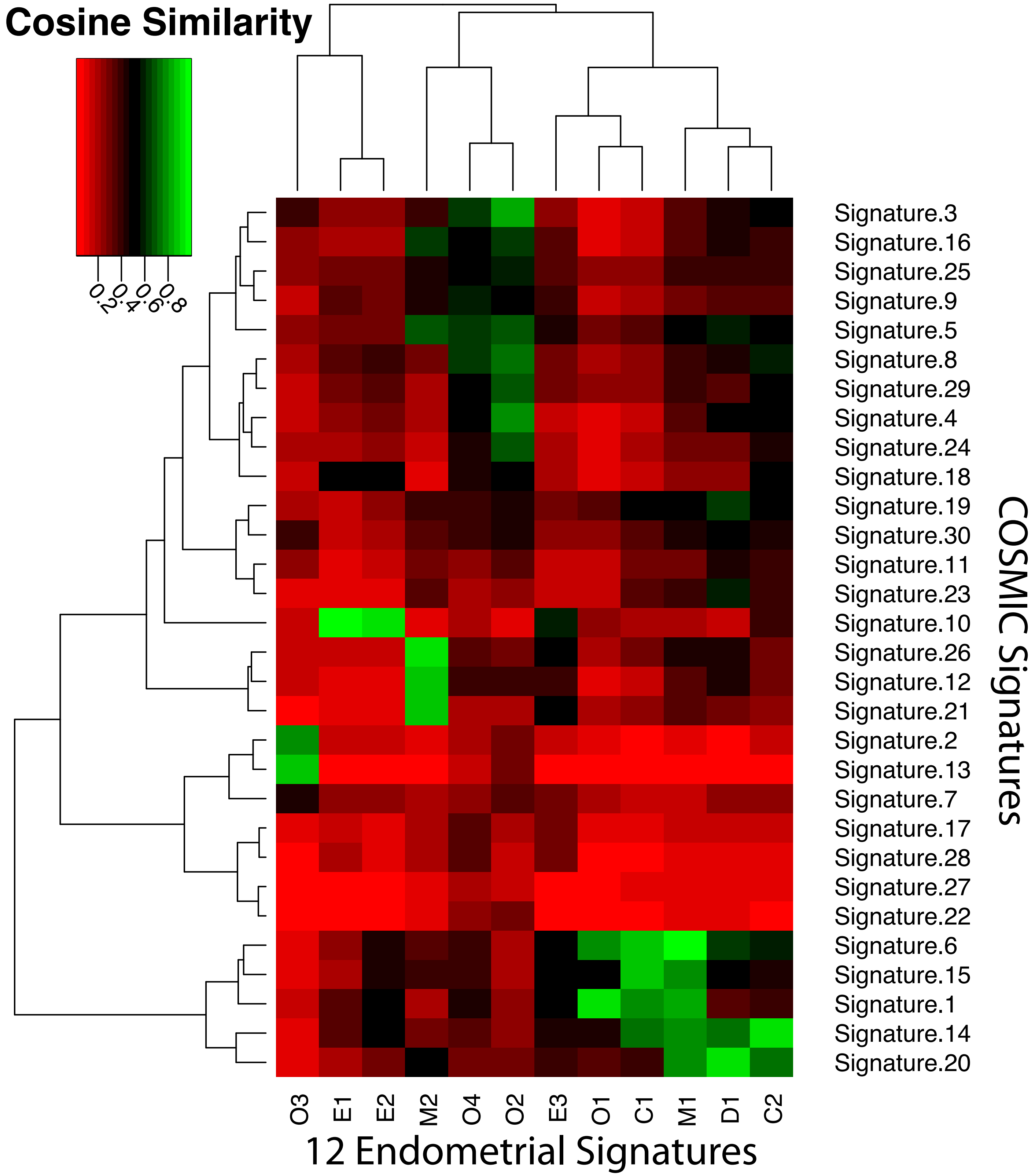

Supplementary Figure 5. Comparison of the twelve mutational signatures identified in this analysis of the TCGA endometrial cancer cohort (projected on to 96 trinucleotide contexts as described in the Methods) with the thirty COSMIC database signatures (http://cancer.sanger.ac.uk/cosmic/signatures). Color represents cosine similarity.

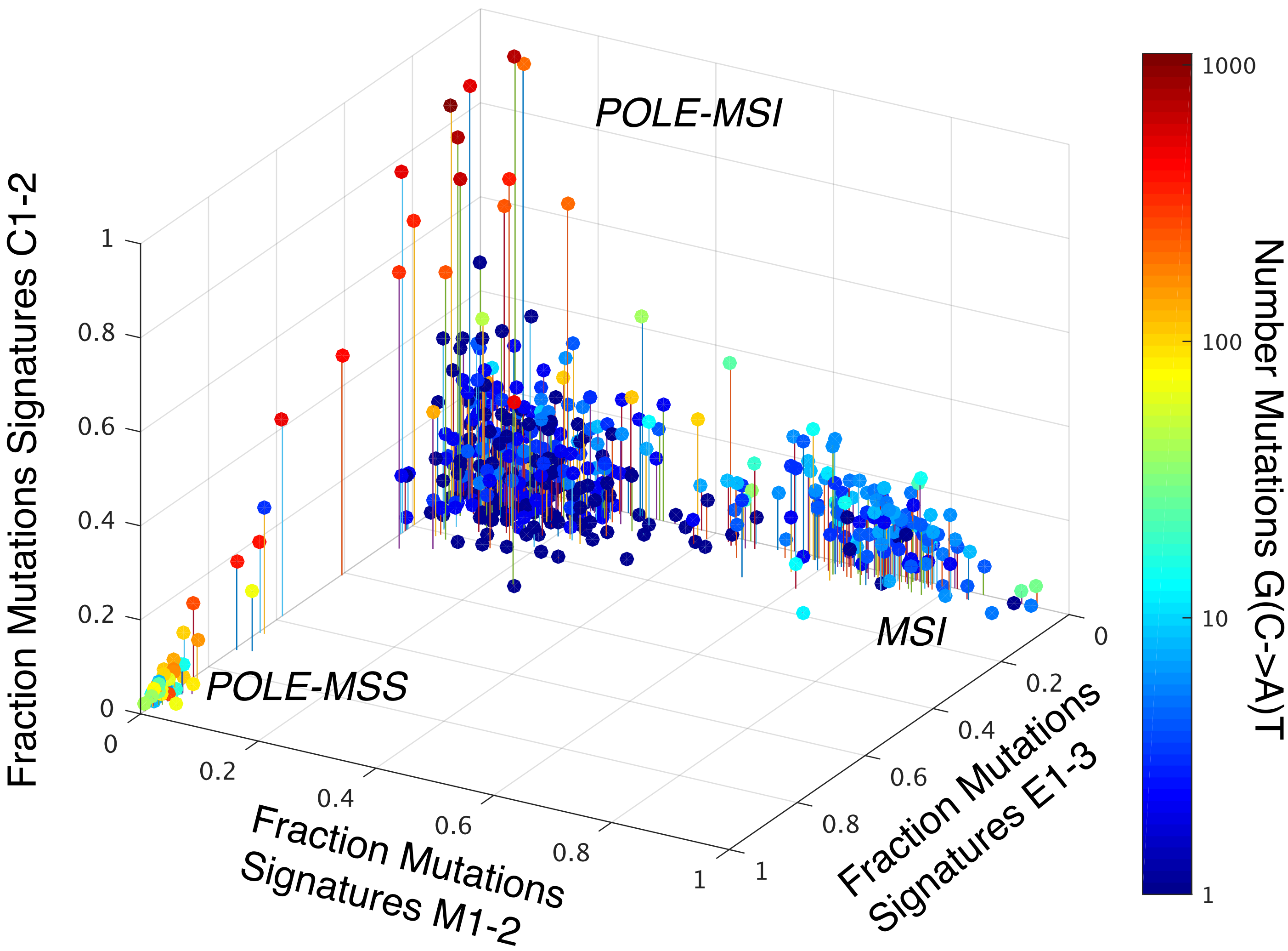

Supplementary Figure 6. Demonstration of the diagnostic G(C>A)T peak. All 531 endometrial samples are plotted in three dimensions by the fraction of mutations contributed by Signatures E1-3, M1-2, and C1-2. Samples are colored by number of C>A mutations in the GCT context. Only samples with predominantly Signature C, i.e. POLE-MSI samples, have substantial amounts of this class of mutations.

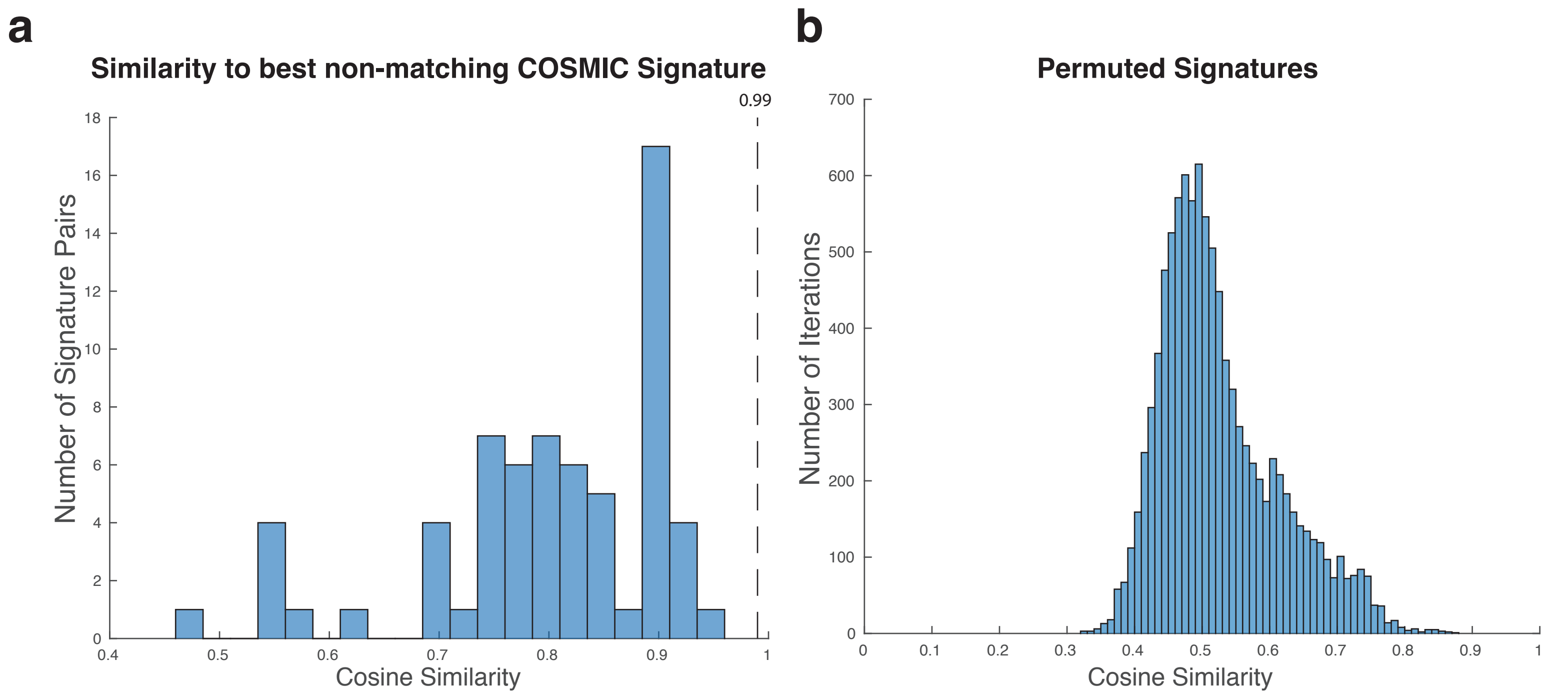

Supplementary Figure 7. False discovery analysis of matching linear combinations of two identified signatures to any single signature from the COSMIC database. (a) The distribution of cosine similarity of matching each pair of our 12 signatures (collapsed to the 96 possible trinucleotide changes) with the closest of 30 COSMIC signatures, excluding the true positive matches (e.g. our Signature O1 plus any signature compared to COSMIC Signature 1) detailed in Supplementary Data 4. Of the remaining comparisons, none had cosine similarity ≥0.99, as was observed between Signatures C1 and C2 with COSMIC Signature 14. (b) To estimate the false discovery rate due solely to mathematical overfitting (i.e., ignoring commonalities in underlying biology between signatures), we shuffled the weights of each of our signatures (collapsed to the 96 possible trinucleotide changes) and calculated the cosine similarity of the best match to the COSMIC database. None of 10,000 trials had a cosine similarity ≥0.9, suggesting at least some degree of shared biology is necessary for stronger similarities.

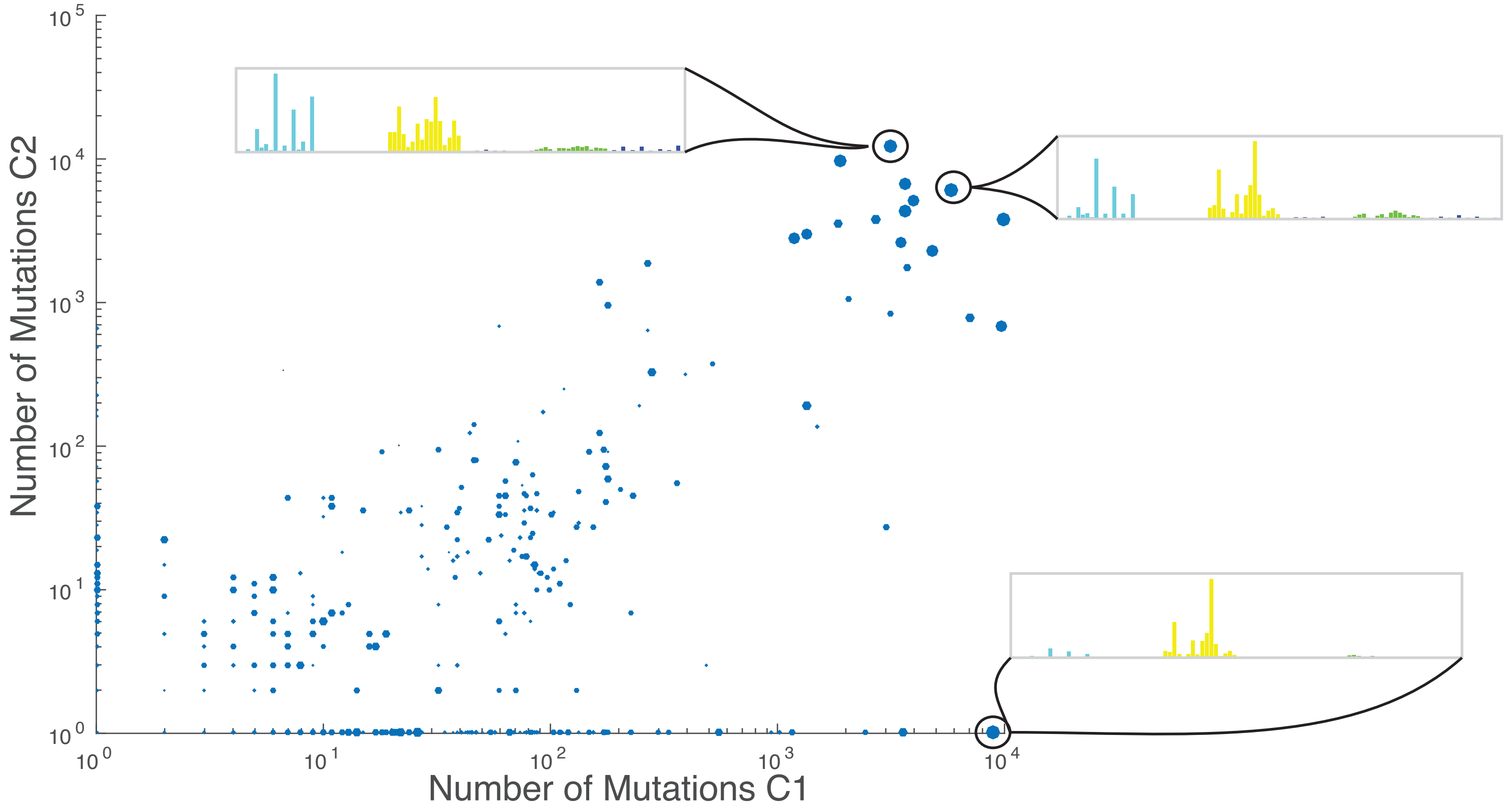

Supplementary Figure 8. Patient-level comparison of the activities of Signatures C1 and C2 for all 531 Endometrial samples. The size of each point represents the summed fraction of mutations assigned to Signatures C1 and C2 in each sample. While the presence of Signature C2 is always accompanied by presence of Signature C1, individual tumors vary significantly in the relative contribution of Signature C1 and Signature C2. Most notably, there are a subset of tumors with a strong contribution from Signature C1 with minimal contribution from Signature C2 (i.e., the samples along the x-axis).

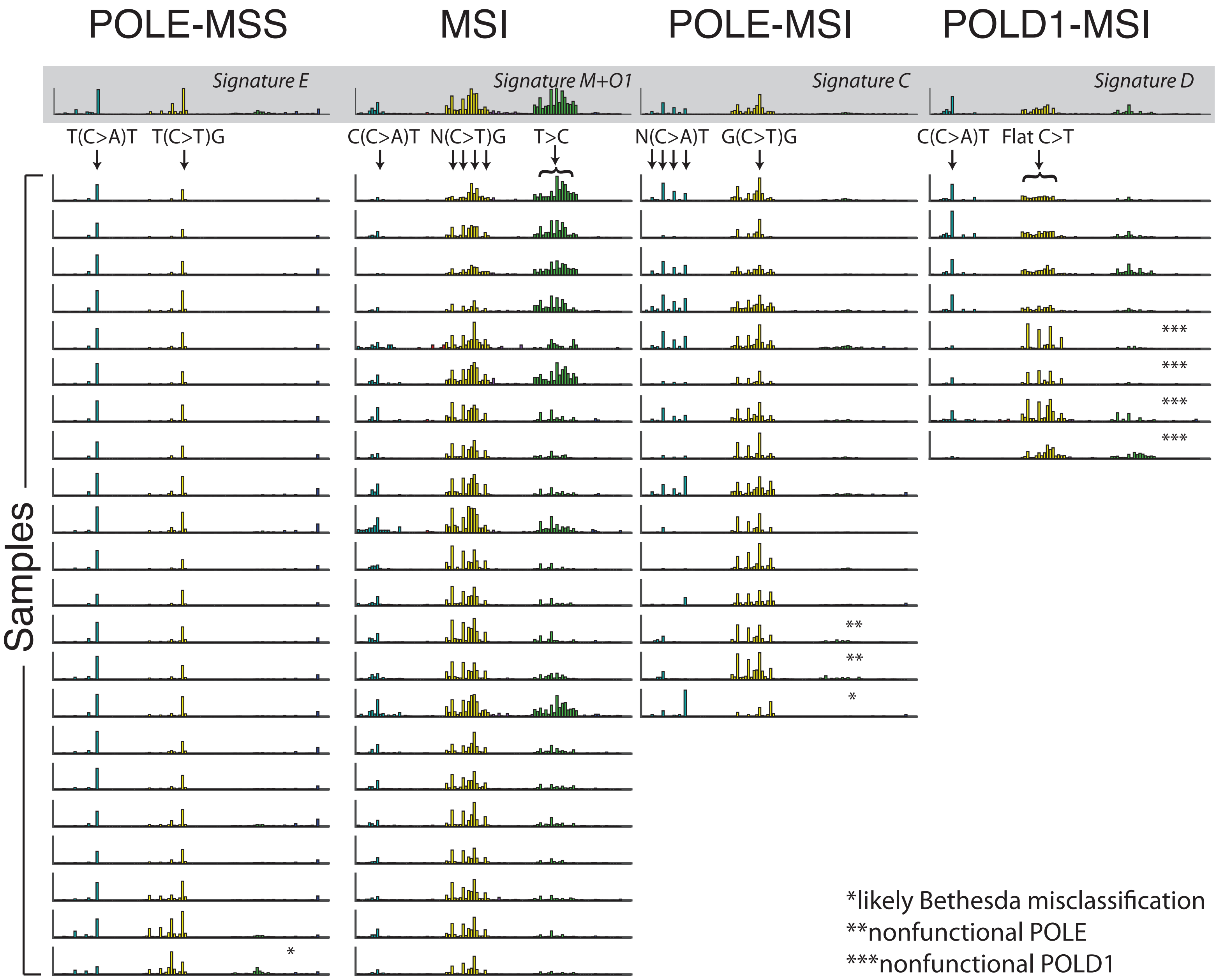

Supplementary Figure 9. Mutational spectra of individual tumors with POLE/POLD1 mutations and/or MMR deficiency. The corresponding signatures described here are shown above (groups with multiple signatures are averaged, e.g. “Signature C” represents equal parts C1 and C2). Characteristic mutational contexts of each group are shown with arrows. The spectra of patients within each class are extremely consistent. Exceptions due to likely nonfunctional polymerase mutations or Bethesda misclassification are highlighted with asterisks. The spectra of the POLE-MSI tumors do not resemble the sum of the POLE-MSS plus MSI tumor spectra. Similarly, the spectra of the POLD1-MSI tumors do not resemble the MSI spectra (no POLD1-MSS cases were present in the cohort). All POLE-MSS, POLE-MSI, and POLD1-MSI cases are shown whereas only the first 22 MSI cases (with WT POLE/POLD1) are shown (a total of 137 cases were present in the dataset).

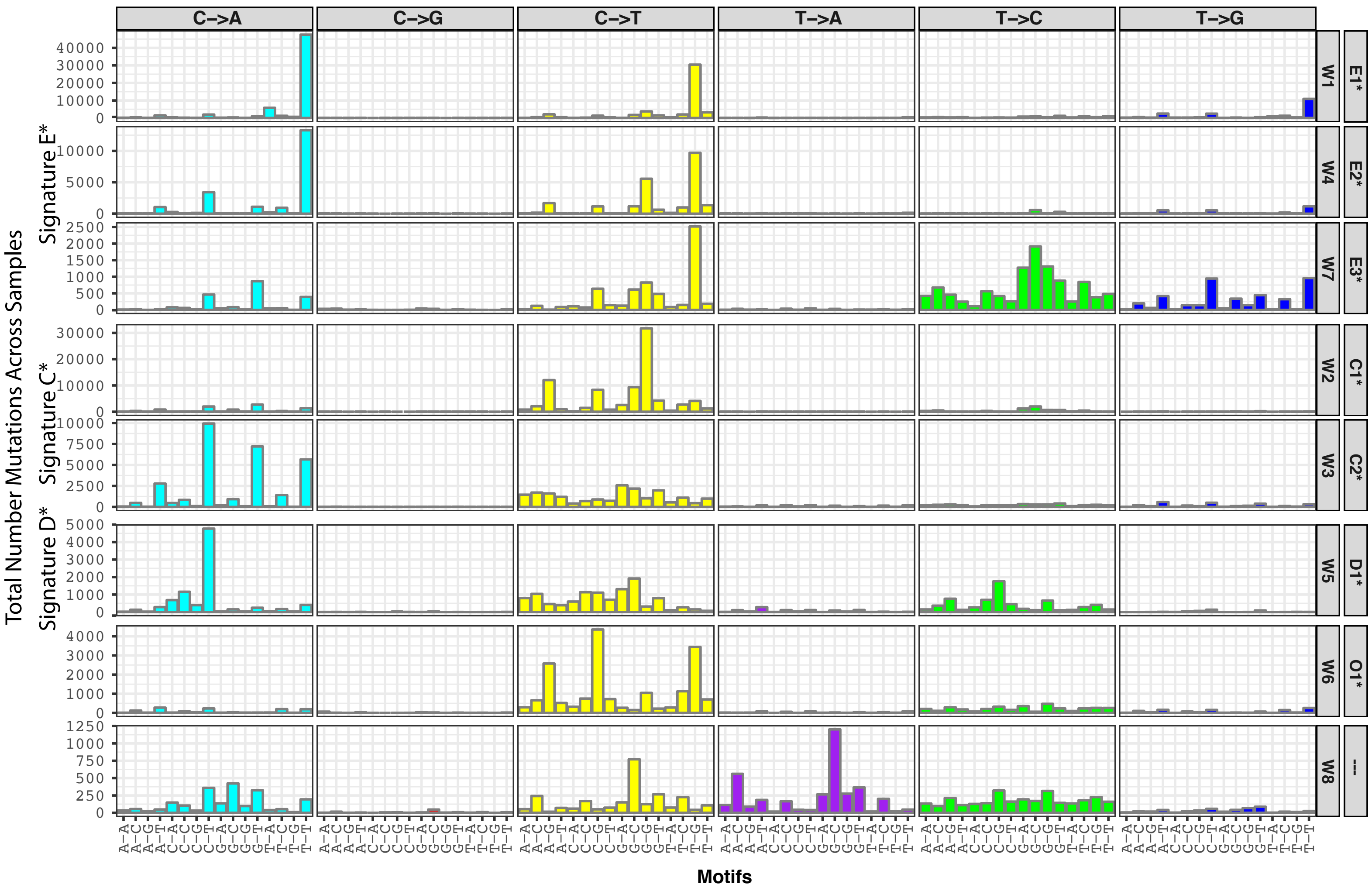

Supplementary Figure 10. Mutational signatures identified by NMF in a cohort of 10 bMMRD GBM samples from Shlien et al. 2015 combined with 59 Endometrial cancers with contributions from Signatures E1-3, C1-2 or D (defined as in Supplementary Data 6b). Seven of the eight signatures correspond to signatures extracted in the analysis of the 531 endometrial samples. Signature W8 was unique to one POLD1-exo* bMMRD GBM sample.

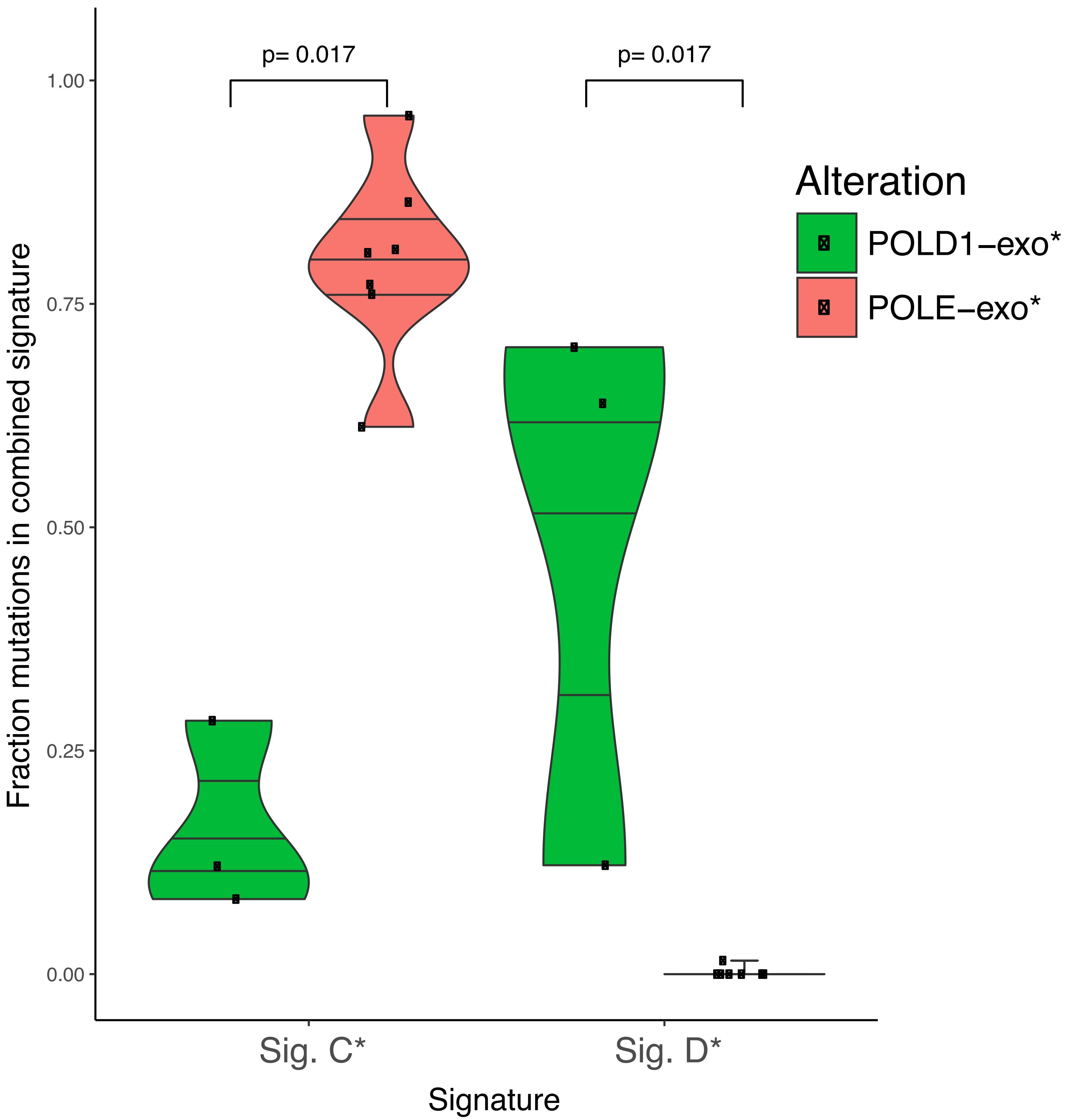

Supplementary Figure 11. Contribution of Signatures C* and D* to total mutational burden in bMMRd tumors with POLE or POLD1 exonuclease domain mutations. POLE-exo* samples display high contributions of Signature C* while POLD1-exo* samples display high levels of Signature D*. Signature C* includes contributions from both Signatures C1* and C2*.

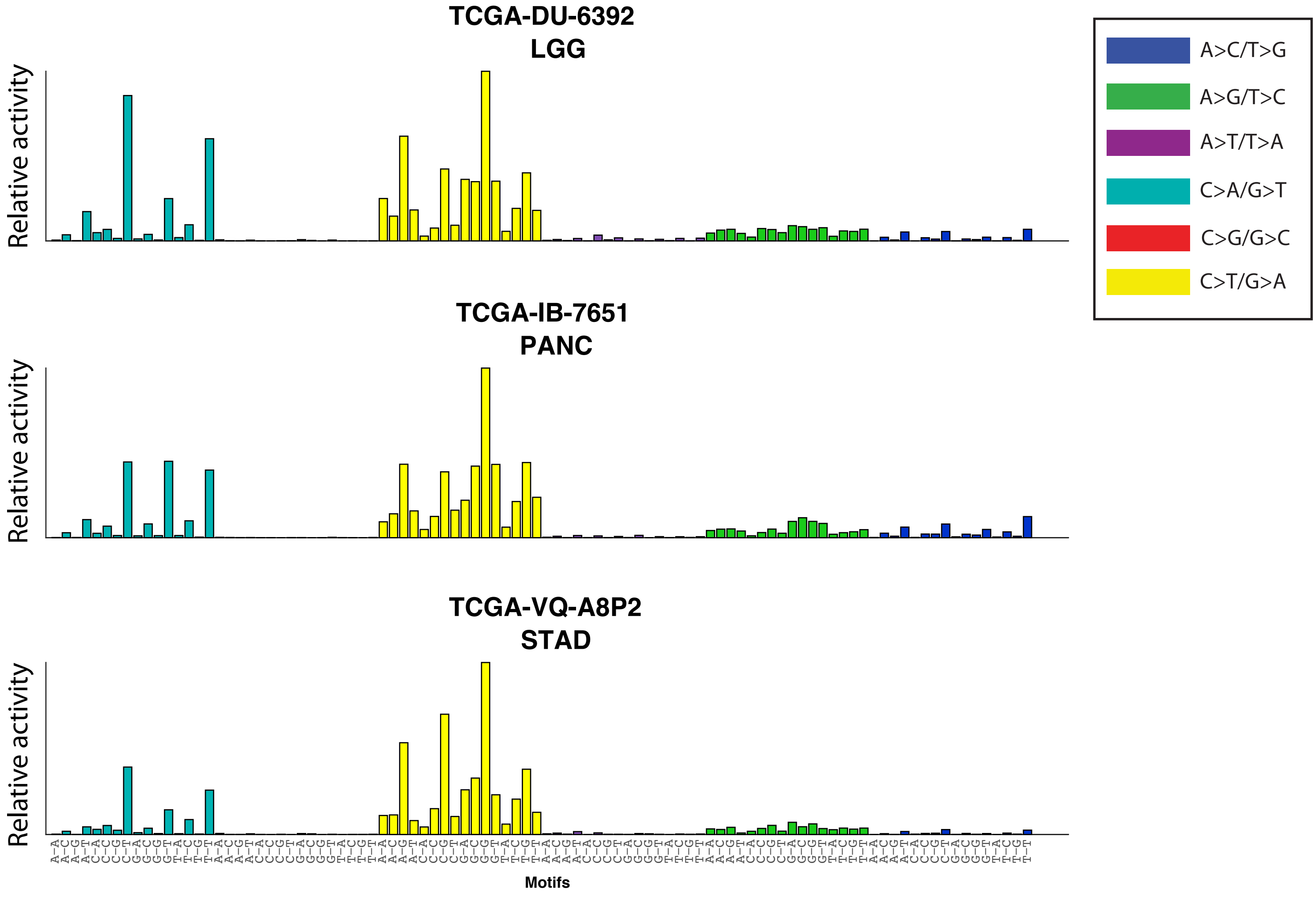

Supplementary Figure 12. Overall mutational spectra of three non-endometrial TCGA tumors with mutational contributions from Signatures C1/2.

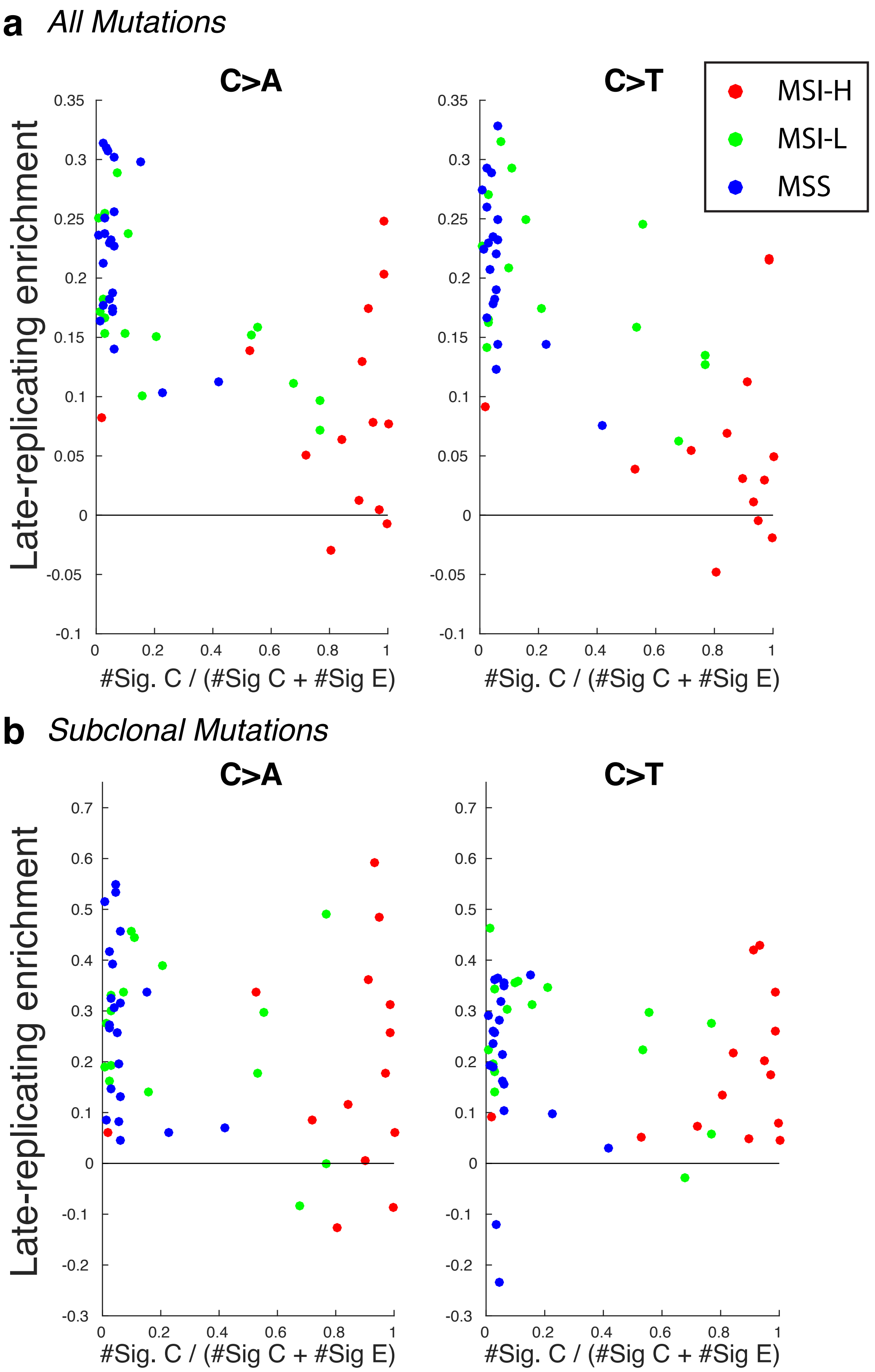

Supplementary Figure 13. The impact of Signature E contributions on the replication timing profiles shown in Figure 5a using both (a) all mutations and (b) subclonal mutations. Samples with POLE-exo* mutations are color-coded by Bethesda MSI status and plotted by the fraction of mutations in the combined POLE-associated signatures (Signatures C plus E) that are contributed by Signature C (i.e., samples with x~0 have no contributions from Signature C and samples with x~1 have no contributions from Signature E). Late-replicating enrichment of C>A and C>T mutations (y-axis) is reduced in POLE-MSI samples (i.e., samples on right side of each plot with x~1) compared to POLE-MSS samples (lefthand side of each plot, x~0). However, even in samples with nearly complete enrichment of Signature C, there is a positive replication-timing slope (y-value >0). As subclonal mutations should be those accumulated after both events are acquired, these should constitute pure Signature C without contamination from early Signature E accumulation.

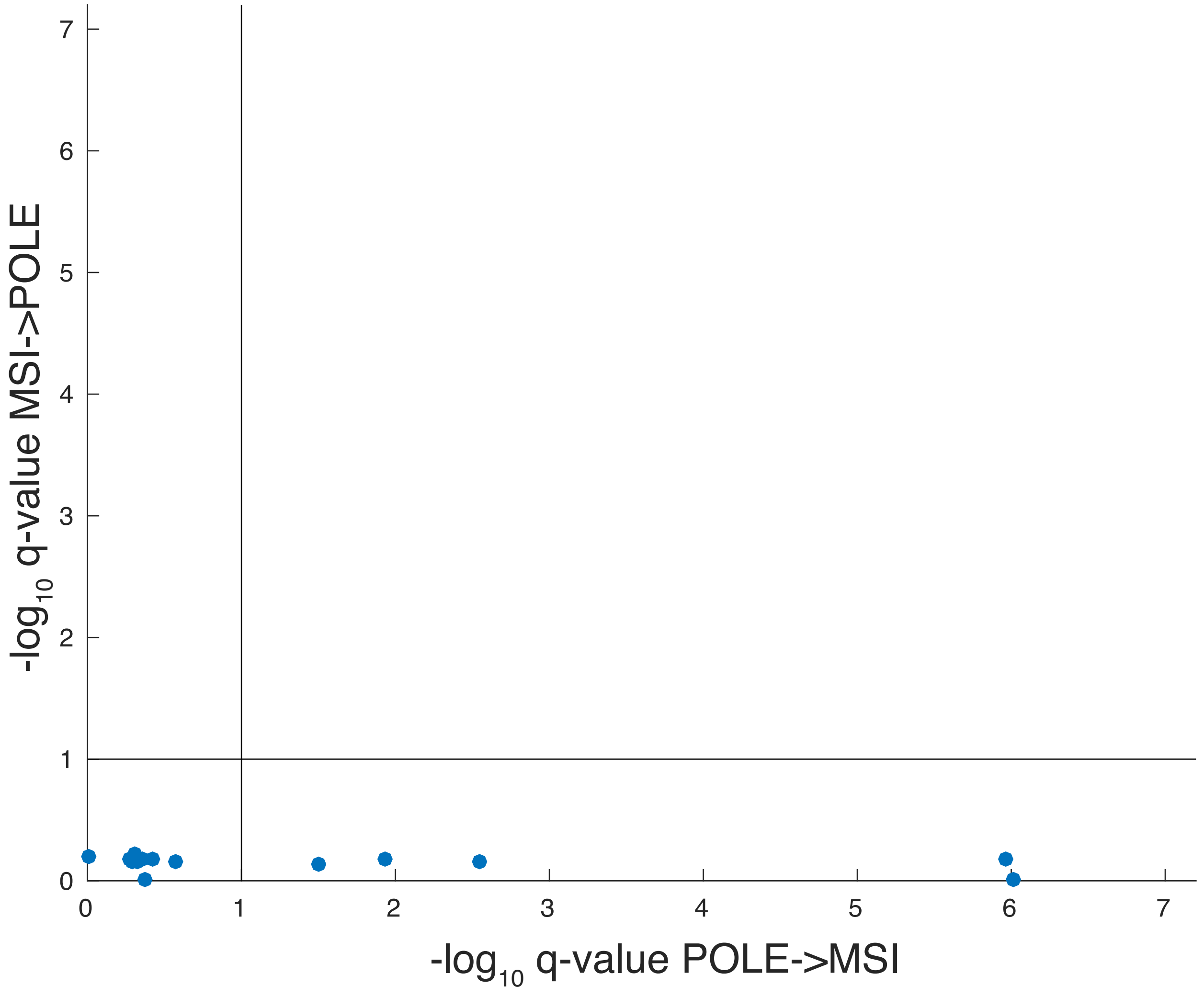

Supplementary Figure 14. False discovery rate (FDR)-corrected q-values for testing the order of the POLE-exo* and MMR deficiency events. The FDR for POLE followed by MSI was <0.1 for 5 tumors. No tumors had a FDR<0.1 for MSI followed by POLE-exo*.
