# Supplementary material for: Distinct mutational signatures characterize concurrent loss of polymerase proofreading and mismatch repair
Source: Nat Commun. 2018 May 1;9:1746. doi: 10.1038/s41467-018-04002-4 (PMC5931517; doi:10.1038/s41467-018-04002-4)
Supplement: Supplementary file 3 — Description of Additional Supplementary Files [file 41467_2018_4002_MOESM3_ESM.pdf]

Description of Additional Supplementary Files

Supplementary Data 1.

A list of all 531 TCGA endometrial tumors that form our primary dataset. Each sample is annotated with relevant genomic attributes and activities of each signature reported here.

Supplementary Data 2.

A summary table of all samples analyzed in this study.

Supplementary Data 3.

The relative contribution of each of the 1544 mutation types to each signature extracted here (the “W” matrix).

Supplementary Data 4.

Summary of prior works, when applicable, characterizing the signatures extracted in this study.

Supplementary Data 5.

List of three non-endometrial TCGA samples with signatures of *POLE*-MSI.

Supplementary Data 6.

Criteria used for signature-based classification of samples.

Supplementary Data 7.

Order of events statistics for five samples in which *POLE* mutations were found to precede MMR lost.
